# A randomized, double-blind, placebo-controlled trial of oral montelukast in acute asthma exacerbation

**DOI:** 10.1186/1471-2466-13-20

**Published:** 2013-03-28

**Authors:** Ali Bin Sarwar Zubairi, Nawal Salahuddin, Ali Khawaja, Safia Awan, Adil Aijaz Shah, Ahmed Suleman Haque, Shahid Javed Husain, Nisar Rao, Javaid Ahmad Khan

**Affiliations:** 1 Section of Pulmonary and Critical Care Medicine, Department of Medicine, The Aga Khan University Hospital, Stadium Road, P.O. Box 3500, Karachi, 74800, Pakistan; 2 Medical College, The Aga Khan University Hospital, Stadium Road, P.O. Box 3500, Karachi, 74800, Pakistan

**Keywords:** Asthma attack, Leukotriene receptor antagonist, Montelukast, PEF, FEV_1_

## Abstract

**Background:**

Leukotriene receptor antagonists (LTRAs) are well established in the management of outpatient asthma. However, there is very little information as to their role in acute asthma exacerbations. We hypothesized that LTRAs may accelerate lung function recovery when given in an acute exacerbation.

**Methods:**

A randomized, double blind, placebo-controlled trial was conducted at the Aga Khan University Hospital to assess the efficacy of oral montelukast on patients of 16 years of age and above who were hospitalized with acute asthma exacerbation. The patients were given either montelukast or placebo along with standard therapy throughout the hospital stay for acute asthma. Improvements in lung function and duration of hospital stay were monitored.

**Results:**

100 patients were randomized; their mean age was 52 years (SD +/− 18.50). The majority were females (79%) and non-smokers (89%). The mean hospital stay was 3.70 ± 1.93 days with 80% of patients discharged in 3 days. There was no significant difference in clinical symptoms, PEF over the course of hospital stay (p = 0.20 at day 2 and p = 0.47 at day 3) and discharge (p = 0.15), FEV_1_ at discharge (p = 0.29) or length of hospital stay (p = 0.90) between the two groups. No serious adverse effects were noted during the course of the study.

**Conclusion:**

Our study suggests that there is no benefit of addition of oral montelukast over conventional treatment in the management of acute asthma attack.

**Trial registration:**

Trial registration number: 375-Med/ERC-04

## Background

Acute asthma accounts for nearly 2 million emergency department visits and 500,000 admissions each year in the US, frequently ranking as a major contributor to time away from work and decreased productivity [1]. Its incidence is on the rise all across the world, especially in the pediatric population, with bronchial asthma accounting for 4% of the pediatric out-patient visits [2,3]. A multi-country survey has demonstrated a rise in asthma symptoms from 8.5% to 11.7% in 13–14 year old Pakistani children, over a 6 year period [4].

Asthma is associated with chronic airway inflammation with recruitment of a number of inflammatory cells including T-cells, mast cells and eosinophils. The macrophages, eosinophils and mast cells in particular have the capacity to synthesize cysteinyl leukotrienes. The interaction of these mediators with the Type 1 cysteinyl leukotriene (CysLT) receptors, located on inflammatory cells and the structural cells of the airways, is implicated in inflammatory cell infiltration, initiation of bronchial smooth muscle contraction, mucus secretion and increased vascular permeability that ultimately leads to airway narrowing [5].

Montelukast is the most commonly used cysteinyl leukotrienes receptor 1 (CysLT-1) antagonist. It has been shown to improve symptoms and lung function (FEV_1_) within 15 minutes of administration in chronic asthma with its effects lasting for a period of at least 24 hours [6].

The existing therapeutic modalities for acute asthma include oxygen and short acting β_2_ agonist bronchodilators in order to promptly reverse airflow obstruction [7]. The systemic corticosteroids are recommended for exacerbations that are unresponsive to initial therapeutic measures but studies have shown a 4–6 hour delay in the onset of the effects of steroid therapy [8]. Such a delay can prove to be catastrophic in the 30% of patients who fail to respond to initial therapy by short acting β_2_-agonists [9]. Furthermore, an increased rate of relapse following an acute exacerbation persists even with corticosteroid therapy [10,11] with an estimated 10% rate of relapse within 7 days of discharge from the emergency room (ER) and a 31% recurrence rate 10 to 21 days after discharge [12-15].

In our study, we tested the hypothesis that treatment with a leukotriene receptor antagonist (LTRA), montelukast sodium would improve airway obstruction and clinical outcomes in acute asthma exacerbation and would subsequently decrease the duration of hospital stay.

## Methods

### Study setting

The patients were identified from the Aga Khan University Hospital (AKUH), located in Karachi, the largest metropolitan city of Pakistan. This is a 650-bed, internationally accredited tertiary care hospital that caters to the needs of a large multi-ethnic urban population. The hospital has a dedicated emergency room (ER) and a 5-bed respiratory special care unit staffed by a team of physicians and nurses trained in the management of respiratory disorders.

### Study subjects

All patients of age 16 and above who presented to the AKUH with acute asthma exacerbation were screened for enrollment in the study. Informed consent was obtained. The eligibility criteria included a diagnosis of acute asthma exacerbation that required hospitalization as defined by the Global Initiative for National Asthma (GINA) Guidelines [7]. The criteria for hospitalization was FEV_1_ < 70% predicted or PEF < 300 L/min after 30 minutes of receiving initial treatment in the ER, respiratory rate > 24 breaths/min and no improvement in symptoms such as shortness of breath or wheezing.

**Figure 1:**
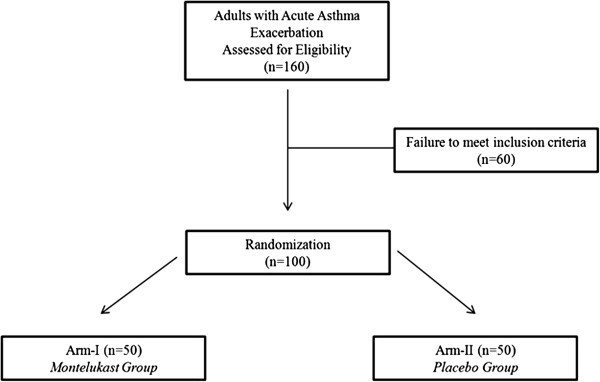
Flow chart of the study.

The patients with the following conditions were excluded from the study; age <15 years, pregnancy, FEV_1_ > 70% predicted or PEF > 300 L/min, a history of tobacco use of >10 years, concomitant therapy with systemic corticosteroids or leukotriene modifiers at any time in the past 4 weeks at the time of admission, any concurrent acute medical condition like myocardial infarction, congestive cardiac failure, diabetic ketoacidosis or shock, acute respiratory failure requiring mechanical ventilation, and improvement in symptoms after being recruited into the study warranting discharge from the emergency department. Patients who were unwilling to consent were also excluded. Flow chart of the study is shown in Figure 1.

### Study design

This was a randomized, double-blind, placebo controlled parallel group drug trial conducted over a period of two years from February 2006 to February 2008. All patients presenting either to the emergency department or outpatient clinics of the AKUH requiring hospitalization with acute asthma exacerbation were screened for inclusion in the study. The study was approved by the Ethical Review Committee (ERC) of the Aga Khan University (375-Med/ERC-04). A written informed consent was obtained before enrollment and findings were shared with subjects interested in the study outcome on clinic follow-up. The patients underwent a baseline spirometry and peak expiratory flow (PEF) testing soon after enrollment. A brief questionnaire was used to obtain information about the duration, severity and treatment of asthma.

### Measurement

The bedside spirometry was done using PiKo-1 (ATS and EU electronic peak flow monitor, Ferraris Respiratory Europe Ltd., Westford SG13 7NW, UK) software which measures the PEF and FEV_1._ The PiKo-1 test was repeated three times. The primary aim of utilizing the software was to facilitate both patient and investigator use. Each test was performed within three minutes of the previous one. The spirometry was done on admission and discharge. Other investigations like chest radiographs and arterial blood gases (ABG’s) were done if deemed necessary by the admitting team.

### Sample size

A group sample size of 50 patients in treatment (Montelukast) and 50 in placebo group achieved 80% power to detect a mean difference of 0.3 liters between the two groups with a mean of 2.2 [16] and standard deviation of 0.7 at 5% significance level using a two-sided two-sample t-test. The required final target sample size was 100 patients.

### Randomization and blinding

The AKUH pharmacy played a pivotal role in the randomization of patients. The trial coordinator at the AKUH pharmacy was the key person to maintain randomization and blinding and was the only one to know the treatment status of the patient. Allocation numbers were generated and assigned to each patient found eligible to be enrolled in the study. Patients were distributed to each arm based on the allocated code. Neither the evaluators, nor the on-call admitting team were made aware of the actual treatment allocations.

The patients were randomly allocated to one of the two study arms; patients in arm-1 received standard therapy and oral montelukast sodium (10 mg once daily) and those in arm-2 received standard therapy along with a placebo.

### Intervention

**Figure 2:**
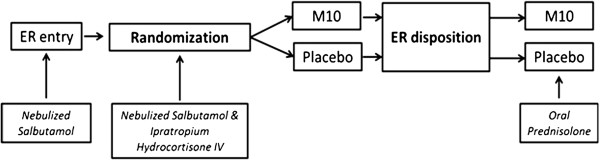
**Trial design.** ER: Emergency room.

All patients with acute asthma underwent clinical assessment for severity of attack and received standard therapy with oxygen and inhaled bronchodilators via jet nebulizer with salbutamol 2.5 mg and ipratropium bromide 500 mcg mixed with 2 cc of normal saline every 15 to 30 minutes. The duration of the high dose bronchodilator therapy was variable (1–4 hours) and subsequently tapered to every 6 hours depending on the symptomatic response to therapy. A dose of systemic corticosteroids in the form of hydrocortisone 200 mg IV was administered in the ER followed by 100 mg every 6 hours. The steroids were subsequently changed to oral prednisolone 0.5 mg/kg/day for 7 days. The trial design followed in the ER is shown in Figure 2.

Additional asthma therapies with aminophylline and magnesium sulphate were given on the discretion of the admitting team when asthma exacerbation was not responding to initial standard therapy after hospitalization. Antibiotics were given only if there was clinical or radiological suspicion of bacterial infection like fever >101 °F, purulent sputum production or clinical or radiological signs of consolidation. The use of antibiotic was not assessed in our study.

Patients in the treatment arm 1 received oral montelukast 10 mg first dose in the ER followed by 10 mg oral dose once a day in evening for the duration of stay in the hospital. The medication was started in the ER as soon as standard therapy was administered, informed consent was obtained and a decision was made for hospitalization.

The placebo was of the same appearance (color and size) and taste as the trial medication; it was prepared by the Hilton Pharma after being approved by Ministry of Health (MoH) Pakistan. The first dose was given in the ER followed by once daily for a period of duration of stay in the hospital. The trial coordinator in AKUH pharmacy managed the storage and distribution of placebo. The serial peak expiratory flow (PEF) monitoring was done; a baseline PEF value was obtained on enrolment, then at 30, 60 and 90 minutes from the baseline value, followed by every 12 hours daily until discharge. A minimum of 3 readings were obtained each time before administration of bronchodilator and the best of 3 was taken as the final value.

### Outcome assessment

The primary outcomes of the study were a) improvement in lung function measured as PEF and FEV_1_ over the course of hospital stay and discharge and b) duration of hospital stay.

Secondary outcome included development of complications such as respiratory failure, cardiac arrest and/or death.

### Statistical analysis

For descriptive analysis mean ± standard deviation are reported for continuous variables, and number (%) for categorical variables. In univariate analyses, differences in proportions for type of treatment groups were assessed by using the Chi-square test or Fisher exact test where appropriate. For contrasts of continuous variables, independent sample t-test was used to assess the difference of means. All analyses were conducted by using the Statistical package for social science (SPSS Release 15.0, standard version, copyright © SPSS; 1989–02), p-values were two sided and considered as statistically significant if < 0.05.

## Results

In total, 160 patients with acute asthma exacerbation were assessed for eligibility. Of the 100 enrolled patients, 50 were randomized to receive oral montelukast. The baseline characteristics of both groups are given in Table 1. Weather change was the major precipitating factor of the acute asthma attack in 51% of the patients followed by infection of the respiratory tract in 45% patients while 57 patients had a positive family history for asthma. The majority of patients were non-smokers, 44 had a prior history of an acute exacerbation and 28 were hospitalized 2 or more times in the past year with 9 requiring ICU admission and 5 requiring intubation and mechanical ventilation as a result of the exacerbation.

**Table 1:** Baseline characteristics of study patients receiving montelukast and placebo (n = 100)

**Number**	**Montelukast**	**Placebo**
	**50**	**50**
Age in years (SD)	50.50 ± 18.26	52.68 ± 18.86
Male: Female (n)	7:43	14:36
Asthma history		
a. Past history of sudden severe exacerbations	22 (44%)	22 (44%)
b. > 2 admissions	17 (34%)	11 (22%)
c. ICU admissions	03 (06%)	06 (12%)
d. Prior intubation	01 (02%)	04 (08%)
Family history of Asthma	30 (60%)	27 (54%)
Medication history		
a) Fluticasone 250 mcg + Salmeterol 25 mcg	24 (48%)	25 (50%)
b) Oral Theophylline	07 (14%)	11 (22%)
c) Inhaled anti-cholinergic	01 (02%)	02 (04%)
Precipitating factors		
a) Infection	23 (46%)	22 (44%)
b) Weather change	27 (54%)	24 (48%)
c) Non-compliance to drugs	01 (02%)	01 (02%)
d) Allergen exposure	07 (14%)	10 (20%)
f) None	04 (07%)	02 (04%)
Smoking status		
a) Non-smoker	47 (94%)	42 (84%)
b) Current smoker	02 (04%)	03 (06%)
c) Ex-smoker	01 (02%)	05 (10%)
Additional therapy		
a) IV aminophylline	03 (06%)	02 (04%)
b) IV magnesium sulphate	16 (32%)	15 (30%)
c) Oral theophylline	10 (20%)	16 (32%)

### Primary outcome measures

**Figure 3:**
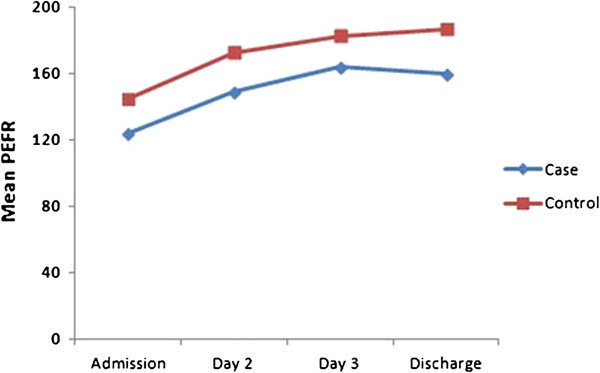
Mean PEF values over the course of hospital stay and discharge.

There was no significant difference in the PEF between both treatment groups during the hospital stay and at discharge (Table 2 and Figure 3). The patients who received montelukast had a mean PEF of 160.12 ± 77.0 L/min while those on placebo had a mean PEF of 187.08 ± 108.9 L/min on discharge (p = 0.15). A similar trend was seen in the FEV_1_ (p = 0.29), where the mean values for the study and placebo groups were 1.07 ± 0.54 L/min and 1.21 ± 0.68 L/min respectively (Table 2).

**Table 2:** **Peak expiratory flow (PEF) & forced expiratory volume in 1 second (FEV**_**1**_**) in patients receiving montelukast vs. placebo**

	**Montelukast**		**Placebo**		**p value**
	**n**	**Mean (±SD)**	**n**	**Mean (±SD)**	
PEF at admission	50	123.82 (±48.76)	50	144.70 (±78.85)	0.11
PEF at day 2	50	149.48 ± 75.76	50	173.41 ± 103	0.20
PEF at day3	50	164.50 ±82.74	50	183.35 ± 113.12	0.47
PEF at discharge	50	160.12 (±77.00)	50	187.08 (±108.93)	0.15
FEV_1_ at admission	50	0.82 (±0.35)	50	0.90 (±0.60)	0.46
FEV_1_ at discharge	50	1.07 (±0.54)	50	1.21 (±0.68)	0.29

There was no significant variation in the duration of hospital stay between both the groups with the mean duration for patients belonging to montelukast and placebo groups being 3.67 ± 1.86 days and 3.72 ± 2.02 days, respectively (p = 0.90).

### Secondary outcome measures

Two patients, one from each arm, developed respiratory failure. No patient in either group was withdrawn due to worsening asthma or adverse drug effect from the study.

## Discussion

Our study did not reveal significant differences in pulmonary function tests measured as FEV_1_ at admission and discharge and PEF measured at specific intervals or length of hospital stay in patients hospitalized with acute asthma exacerbation that were given oral montelukast vs. placebo. The efficacy and tolerability profile of oral montelukast were comparable to placebo and no serious adverse effects were encountered.

The pathology of asthma triggers the arachnoid acid cascade leading to formation of leukotrienes via the 5-lipoxygenase pathway. The cysteinyl leukotrienes possess pro-inflammatory characteristics which can directly cause or potentiate airflow obstruction by increased mucosal secretion and bronchospasm [5,17]. Leukotriene pathway modifiers, hence, are a subject of interest as a possible adjunct therapy in the acute management of asthma exacerbation. However, our results are in contrast to data recently published by Ramsay et al. They randomized 73 patients and found a significantly higher peak expiratory flows (PEFs) measured in the morning after admission in patients who received montelukast (p = 0.046, 95% CI of 1.15-113.6 L/min) as compared to patients who did not [18]. A study by Silverman et al. evaluated the effects of another LTRA, zafirlukast. They randomized patients into three groups; oral zafirlukast at 20 mg and 160 mg vs. placebo. They looked at the time to relapse in the outpatient setting after discharge from the emergency department and found reduction in the absolute rate of relapse by 5.3% in patients treated with zafirlukast. They reported significant improvement in FEV_1_ and dyspnea in the ER only with 160 mg of zafirlukast [19]. Other studies have also looked into effects of intravenous montelukast in managing acute asthma exacerbations. Camargo et al. randomized 201 patients to three groups with two receiving separate doses of montelukast (7 mg and 14 mg) and one group receiving placebo. They reported significantly higher FEV_1_ in patients receiving standard therapy with montelukast as compared to placebo at 10 minutes (p = 0.03), 20 minutes (p = 0.007) and two hours (p = 0.003) [20]. These results were validated in a more recent study in Japan which reported both IV monteleukast 7 mg and 14 mg to be effective as an adjunct therapy over 60 minutes; p < 0.05 and p < 0.001 respectively [21].

To the best of our knowledge, our study is the first one to report no added benefits of using montelukast in acute asthma exacerbation in hospitalized adult population. Other studies which report similar findings mostly correspond to the pediatric population [22-24]. Nelson et al. [22] and Morris et al. [23] did not find any significant increase in FEV_1_ by using oral and intravenous montelukast respectively while Todi et al. reported similar proportion of children having Modified Pulmonary Index Score ≤ 9 in both the study and control groups [24].

A possible reason we failed to find significant improvement is that we looked at PEFs early in the course of hospitalization instead of FEV_1_ in comparison to the positive studies cited above. Another reason for failure of significant improvement might be the use of enteral route of administration. However, Dockhorn et al. conducted a study comparing the effectiveness of intravenous montelukast vs. oral montelukast vs. placebo in the setting of acute asthma. Though intravenous montelukast was quicker in onset of action with the mean percentage change in FEV_1_ higher at earlier time intervals (15 mins to 1 hour), this difference decreased over the time and was not significant (p > 0.05). Moreover, there was no difference in mean maximum percentage change in FEV_1_ from baseline between intravenous and oral montelukast (p = 0.071) [6].

This study has several limitations. Firstly, our study sample is relatively small. Secondly, we excluded patients with respiratory failure requiring positive pressure ventilation, either noninvasive or invasive. Both these factors may have impacted on the strength of difference observed in the two groups. Another limitation is the lack of biological surrogate markers like cysteinyl leukotrienes levels which have been shown to be higher in states of acute asthma exacerbation [25]. It is possible that these levels may have reduced in the patients but did not translate into clinical effectiveness yet. The use of Piko-1 pocket spirometer is another limitation in our study. A study from Switzerland revealed that the accuracy of Piko-1 spirometer is acceptable. However, it tended to underestimate FEV1 in the lower range in 20 volunteers [26]. Lastly, this was a single center study and hence, cannot be generalized to the whole population.

## Conclusion

Our study suggests that there is no added benefit of using montelukast along with the standard therapy for the management of acute asthma exacerbation in hospitalized adult population. We recommend that larger scale multicenter trials would better help to evaluate the role of cysteinyl leukotrienes antagonists in treating acute exacerbations of asthma.

## Abbreviations

LTRAs: Leukotriene receptor antagonists; PEF: Peak expiratory flow; FEV1: Forced expiratory volume in 1 second; AKUH: Aga Khan University Hospital; ER: Emergency room

## Competing interests

The authors declare that they have no competing interest.

## Authors’ contributions

The proposal was prepared by ABSZ in consultation with NS, ASH, SJH, NR and JAK. NS, ASH, AK and AAS have analyzed and interpreted the patient data. SA assisted in statistical analysis and manuscript writing. ABSZ, NS, AK and AAS were major contributors in writing the manuscript. All authors read and approved the final manuscript.

## Pre-publication history

The pre-publication history for this paper can be accessed here:

http://www.biomedcentral.com/1471-2466/13/20/prepub
